# Identification of ARF transcription factor gene family and its defense responses to bacterial infection and salicylic acid treatment in sugarcane

**DOI:** 10.3389/fmicb.2023.1257355

**Published:** 2023-09-07

**Authors:** Jia-Xin Lin, Ahmad Ali, Na Chu, Hua-Ying Fu, Mei-Ting Huang, Sylvain Ntambo Mbuya, San-Ji Gao, Hui-Li Zhang

**Affiliations:** ^1^National Engineering Research Center for Sugarcane, Fujian Agriculture and Forestry University, Fuzhou, China; ^2^Faculté des Sciences Agronomiques, Département de production végétale, Laboratoire de Recherche en Biofortification, Defense et Valorisation des Cultures (BioDev), Université de Lubumbashi, Lubumbashi, Democratic Republic of the Congo

**Keywords:** auxin response factor, biotic and abiotic stress, expression pattern, sugarcane, stress response

## Abstract

Auxin response factor (ARF) is a critical regulator in the auxin signaling pathway, involved in a variety of plant biological processes. Here, gene members of 24 *SpapARFs* and 39 *SpnpARFs* were identified in two genomes of *Saccharum spontaneum* clones AP85-441 and Np-X, respectively. Phylogenetic analysis showed that all *ARF* genes were clustered into four clades, which is identical to those *ARF* genes in maize (*Zea mays*) and sorghum (*Sorghum bicolor*). The gene structure and domain composition of this ARF family are conserved to a large degree across plant species. The *SpapARF* and *SpnpARF* genes were unevenly distributed on chromosomes 1–8 and 1–10 in the two genomes of AP85-441 and Np-X, respectively. Segmental duplication events may also contribute to this gene family expansion in *S. spontaneum*. The post-transcriptional regulation of *ARF* genes likely involves sugarcane against various stressors through a miRNA-medicated pathway. Expression levels of six representative *ShARF* genes were analyzed by qRT-PCR assays on two sugarcane cultivars [LCP85-384 (resistant to leaf scald) and ROC20 (susceptible to leaf scald)] triggered by *Acidovorax avenae* subsp. *avenae* (*Aaa*) and *Xanthomonas albilineans* (*Xa*) infections and salicylic acid (SA) treatment. *ShARF04* functioned as a positive regulator under *Xa* and *Aaa* stress, whereas it was a negative regulator under SA treatment. *ShARF07/17* genes played positive roles against both pathogenic bacteria and SA stresses. Additionally, *ShARF22* was negatively modulated by *Xa* and *Aaa* stimuli in both cultivars, particularly LCP85-384. These findings imply that sugarcane *ARFs* exhibit functional redundancy and divergence against stressful conditions. This work lays the foundation for further research on *ARF* gene functions in sugarcane against diverse environmental stressors.

## 1. Introduction

Auxin is one of the crucial plant hormones participating in various biological processes such as plant growth and development as well as stress responses (Yu et al., 2022). Auxin response factors (ARFs), which are a key component of auxin signaling, function as an important transcription factor (TF) by binding directly to the auxin response elements (AuxREs: TGTCNN) in the promoters of primary/early auxin response genes (Yu et al., 2022; Rienstra et al., 2023). Most ARF proteins contain the N-terminal B3 type DNA-binding domain (DBD), the carboxyl (C)-terminal dimerization domain (CTD), and the non-conserved middle region (MR) located between the DBD and CTD (Song et al., 2023). The DBD of an ARF binds specifically to AuxREs in promoters of targeted genes to activate or repress transcriptional expression, while variations in the two last nucleotides of AuxREs (TGTCAT, TGTCAC, and TGTCGG) affect the interaction between ARFs and AuxREs and thus fine-tune the transcriptional response profile of downstream genes (Yu et al., 2022; Rienstra et al., 2023; Song et al., 2023). The CTD of an ARF includes the Phox and Bem1p (PB1) regions for dimerization, which is like the III and IV elements of Aux/IAA, facilitating protein–protein interaction through homodimerization of ARF proteins or heterodimerization of ARF and Aux/IAA proteins (Li et al., 2023). The MR of an ARF with variable sequence length and composition functions as an activation domain (AD) or repression domain (RD) (Li et al., 2023; Song et al., 2023).

The ARF gene family has been examined in several plant taxa, including flowering plants, bryophytes, and lycophytes, but *ARF* gene numbers are highly variable among plant species, ranging from 3 genes in *Marchantia polymorpha* to 51 genes in *Glycine max* (Chandler, 2016; Li et al., 2016, 2023). Recently, a total of 112 *ARFs* were identified in five genomes of orchid species, namely, *Apostasia shenzhenica* (17 *AsARFs*), *Dendrobium catenatum* (21 *DcARFs*), *Phalaenopsis aphrodite* (16 *PaARFs*), *Phalaenopsis equestris* (34 *PeARFs*), and *Vanilla planifolia* (24 *VpARFs*) (Bai et al., 2023). Sixty-seven *TaARFs* were identified in bread wheat (*Triticum aestivum*), distributed across six clades (Chaudhary et al., 2023). In addition to plant growth and development, increasing studies have shown that *ARF* family genes play a significant role in biotic and abiotic stress responses in many plants (Bouzroud et al., 2018; Li et al., 2023; Song et al., 2023).

Numerous ARF family genes are involved in plant defense responses to diverse environmental stresses. For example, transcript levels of most *SlARF* genes were deceased in tomato (*Solanum lycopersicum*) leaves exposed for 6 h to *Pseudomonas* strains including *P. putida*, *P. fluorescens*, or *P. syringae* DC3000 (Bouzroud et al., 2018). Rice *OsARF17* played a key role in plant defense against different types of plant viruses such as fijiviruses, tenuivirus, and cytorhabdovirus through these viral proteins manipulating *OsARF17* in distinct modes (Zhang et al., 2020). When leaves of two wheat cultivars with contrasting resistance to rust were infected with *Puccinia triticina*, distinct expression patterns of five *ARF* genes (*TaARF4/6/12/16/27*) demonstrated their opposite roles in incompatible (R-Pathogen) and compatible (S-Pathogen) interactions in response to this pathogen infection (Chandra et al., 2020). *BdARF4* and *BdARF8* played positive roles in response to salicylic acid (SA) and heavy metals in *Brachypodium distachyon* (Liu N. et al., 2018). *S1ARF8A* and *S1ARF10A* as well as their specific miRNA were involved in the regulation of salt and drought responses (Bouzroud et al., 2018), while *SlARF4* was regulated by osmotic and salt stress in tomato (*S. lycopersicum*) (Bouzroud et al., 2020). The expression levels of *TaARF8*, *TaARF9*, and *TaARF21* were significantly altered by low temperatures in wheat (*T. aestivum*) (Xu et al., 2020). *AcARF1a* and *AcARF10* were negatively responsive to SA stress in kiwifruit (*Actinidia chinensis*) (Su et al., 2021). Three *ClARF* genes (*ClARF4/8/12*) were negatively regulated in response to abscisic acid (ABA) and SA treatments, while *ClARF20* was positively regulated under Indole-3-acetic acid (IAA) and SA treatments (Zhai et al., 2023).

Modern sugarcane cultivars (*Saccharum* spp. hybrid, 2n = 100–130) are highly polyploids and aneuploids derived from interspecific hybridization between *S. officinarum* (2n = 8x = 80) and *S. spontaneum* (2n = 4x–16x = 40–128) (Ali et al., 2019; Zhang et al., 2022). Moreover, *S. spontaneum* exhibits three basic chromosome numbers (x = 8, 9, or 10) (Zhang et al., 2022). The high variation in ploidy levels of *S. spontaneum* genomes contributes to the study of the evolution of autopolyploidy genomes in plants (Piperidis and D’Hont, 2020; Zhang et al., 2022). The two tetraploid genomes of *S. spontaneum* clones AP85-441 (1n = 4x = 32, x = 8) and Np-X (2n = 4x = 40, x = 10) have been published (Zhang et al., 2018, 2022), which provides a powerful resource to identify many gene families. Our previous studies revealed that the SA-mediated signaling pathways played essential roles in sugarcane resistance to infection by *Xanthomonas albilineans* (*Xa*) causing leaf scald (Zhao et al., 2022) and *Acidovorax avenae* subsp. *avenae* (*Aaa*) causing red stripe (Chu et al., 2022; Zhao et al., 2023). However, the genome-wide characterization of *ARFs* and their expression profiles in sugarcane in response to biotic and abiotic stimuli remains unclear. Thus, the objective of this study was to systematically characterize ARF family genes from the polyploid *S. spontaneum* and their transcript expression profiles in sugarcane cultivars under *Aaa* and *Xa* infections as well as SA treatment, which provides an important clue for the subsequent functional identification of *ARFs* in sugarcane.

## 2. Materials and methods

### 2.1. Sequence retrieval of ARF gene family in *Saccharum spontaneum* genome

The genome sequences for *S. spontaneum* AP85-441 were obtained from The Ming Laboratory^1^ and those for *S. spontaneum* Np-X were obtained from the Zhang Laboratory.^2^ The BLASTp algorithm was used to search for the genes of *SpapARF* from AP85-441 and *SpnpARF* from Np-X and to verify the highest number of genes. The 35 maize *ARF* gene sequences (Liu et al., 2011) were acquired from the Maize Genome database^3^ and used as bait in a BLASTp search (score value: ≥ 100 and e-value ≤1 × 10^−5^). The Pfam database, which is accessible online at http://pfam.sanger.ac.uk/ was searched for the Hidden Markov Model (HMM) file corresponding to the ARF domain (PF06507). HMMER3.0 was used to retrieve *ARF* genes from the *S. spontaneum* clones AP85-441 and Np-X genome databases. False sequences were manually eliminated. The sequences of candidate genes were further confirmed by finding their functional domains through the CDD tool in NCBI,^4^ Pfam,^5^ and SMART.^6^ Based on more than 90% resemblance and a high bit score with identified *ARF* genes, these *SpapARF* and *SpnpARF* genes were further validated in two transcriptome datasets (Bio-project numbers: PRJNA579959 and PRJNA549590).

### 2.2. Prediction of physiochemical properties and identification of orthologous gene clusters

The ExPASy Server^7^ was used to determine the properties of the inferred ARF proteins, such as isoelectric points. The Sequence Manipulation Suite^8^ was used to calculate molecular weights, and the WoLF PSORT server^9^ was used to predict subcellular localization. The orthologous ARF proteins in *S. spontaneum* clones (AP85-441 and Np-X), *Z. mays*, and *S. bicolor* were discovered through orthovenn2^10^ based on the whole genome protein sequences of these species.

### 2.3. Prediction of gene structure and conserved motif

The coding sequences (CDS) and protein sequences of identified *ARF* genes were utilized to construct the gene structure (exon-intron distribution) using the online GSDS.^11^ Protein sequences of ARFs were used in MEME Suite^12^ with default parameters to identify the conserved motifs and then visualized by TBtools v1.120 (Chen et al., 2020).

### 2.4. Multiple sequence alignments, phylogenetic, and *Cis*-regulatory analysis

Multiple sequence alignments of all identified ARF proteins were performed by the ClustalW algorithm implemented in the MEGA6.0 program with default parameters (Kumar et al., 2018). The reference protein sequences of ARF gene family were downloaded from *Z. mays* (Zm)^13^ (Liu et al., 2011; Xing et al., 2011), *Fagopyrum tataricum* (Ft) (see text footnote 3) (Liu M. et al., 2018), and *S. bicolor* (Sb)^14^ (Chen et al., 2019) genome databases. The neighbor-joining method in MEGA6.0 software was used for phylogenetic analysis, and bootstrap analysis with 1,000 replicates was used to determine statistical reliability. The promoter sequences (1.5 kb upstream of the start codon) of *SpapARFs* and *SpnpARFs* were scanned for the available *cis*-elements at PlantCARE.^15^

### 2.5. Chromosomal location, gene duplication, and synteny analysis

The chromosomal location of each ARF family gene was retrieved from the general feature format 3 (gff3) file, and the chromosome number, start position, and end position of each gene were extracted and visualized using Tbtools v1.120 (Chen et al., 2020). We defined the gene duplication in accordance with the following criteria: (Ali et al., 2020) the alignment length covered >90% of the longer gene; (Ali et al., 2019) the aligned region had an identity >90%; (Ali et al., 2022) only one duplication event was counted for tightly linked genes. Comparative synteny analysis was performed to observe the evolutionary genome conservations amongst *S. spontaneum* clones (AP85-441 and Np-X), *Z. mays*, and *S. bicolor*. All these genomes and GFF3 files were scanned using Super-fast McScanX in TBtools, and the generated files were utilized for multiple synteny maps. The duplicated genes were investigated using an MCScanX run on each of the entire *S. spontaneum* genomes.

### 2.6. Prediction of miRNAs targeting *Saccharum spontaneum* ARFs

To understand the underlying regulatory mechanism of miRNAs involved in the regulation of *SpapARF* and *SpnpARF* genes, their CDS sequences were utilized to predict miRNA-targeted sites using the psRNATarget database (available at https://www.zhaolab.org) with the default parameters. Cytoscape software v.3.8.2 (available at https://cytoscape.org/) was operated to generate the schematic diagram depicting the interaction networks between miRNAs and targeted *ARF* genes.

### 2.7. Gene expression profiling analysis using RNA-sequencing datasets

The published RNA-sequencing (RNA-seq) datasets by our research group were used to examine the expression patterns of identified *ARF* genes. One transcriptome dataset (PRJNA579959) was derived from sugarcane cultivars, ROC22 (resistant to red stipe) vs. MT11-610 (susceptible to red stipe) (Chu et al., 2020), and another transcriptome dataset (PRJNA549590) was derived from LCP 85–384 (resistant to leaf scald) vs. ROC20 (susceptible to leaf scald) (Ntambo et al., 2019) under pathogenic bacteria *Aaa* and *Xa* infection, respectively. The FPKM values were calculated for entire *ARF* genes, and the log_2_(Fold Change) transformed values of these *ARFs* were used to generate and display heatmap using TBtools and the Heatmapper tool.^16^ The parameters of |log_2_(Fold Change)| > 1.0 and value of *p* <0.05 were set as the threshold for significantly differential expression.

### 2.8. Stress treatments and qRT-PCR analysis

Two sugarcane cultivars, LCP85-384 and ROC20, were used for stress treatments. For biotic stress, the two bacterial species, *Aaa* strain CNGX08 and *Xa* strain Xa-FJ1, were used to inoculate sugarcane young plants and sampled following the protocols developed by Zhao et al. (2023) and Ntambo et al. (2019), respectively. For SA treatment, the protocol developed by Ali et al. (2020) was used. The Trizol^®^ reagent (Invitrogen, United States) was used to extract total RNA from leaf samples. Recombinant DNase-I (Takara, Dalian, China) was used to eliminate any remaining genomic DNA before further qRT-PCR assays. The first-strand cDNA synthesis kit from Takara (China) was used to synthesize cDNA in accordance with the manufacturer’s instructions. The qRT-PCR was carried out following the protocol developed by Ali et al. (2020). The qPCR program consisted of denaturation at 95°C for 30 s, followed by 40 cycles of 5 s at 95°C and 30 s at 60°C. Three biological and three technical replicates for each sample were performed. Primers for six candidate *ARF* genes (Supplementary Table S1) were designed using the GeneScript^®^.^17^ The glyceraldehyde-3-phosphate dehydrogenase (*GAPDH*) was used as a reference gene and the 2^-ΔΔCt^ quantitative method was applied to determine the relative quantitative mRNA profiling.

### 2.9. Statistical analysis

An analysis of variance (One-way ANOVA) was utilized for different gene expression levels at each time point. Data were gained from three biological replicates, and each biological replicate contains three technical replicates. The least significant difference (LSD) test was applied to analyze mean differences at *p* ≤ 0.05.

## 3. Results

### 3.1. Identification of ARF gene clusters in monocot crops

**Figure 1:**
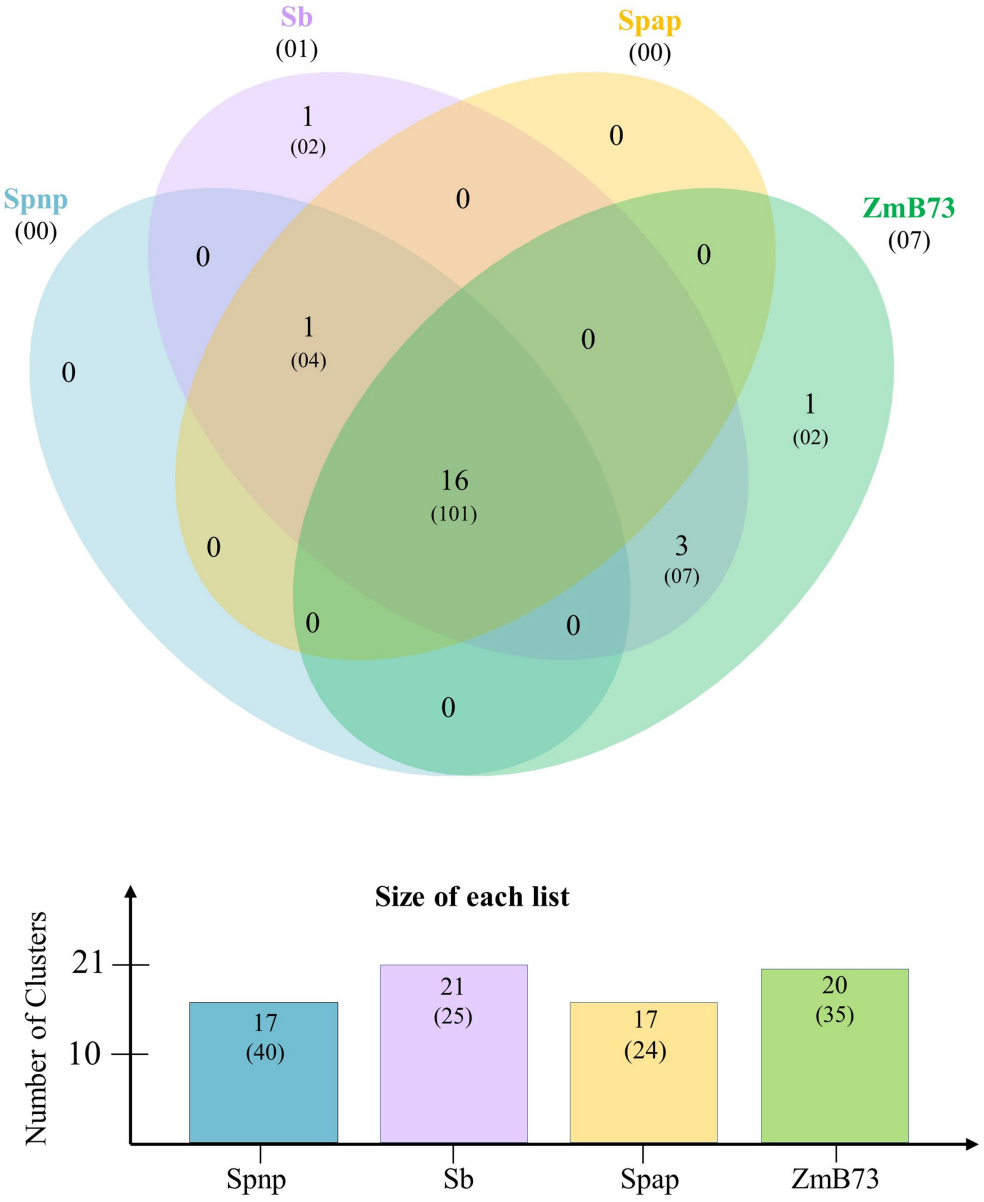
Orthologous ARF family gene clusters among *S. spontaneum* AP85-441 (Spap), *S. spontaneum* Np-X (Spnp), *S. bicolor* (Sb), and *Z. mays* B73 (ZmB73). The number in each sector of the diagram indicates the number of homologous clusters and the numbers in parentheses indicate the total number of genes contained within the associated clusters. The numbers in parentheses below the species names indicate the number of species-specific singletons (genes with no homologs).

Comparative genome analysis was conducted to explore the gene space of *S. spontaneum* clones AP85-441 and Np-X together with *S. bicolor* and *Z. mays* B73 (Supplementary Figure S1). The maximum clusters of 35,866 (123,166 proteins) were recorded in Np-X, followed by 31,332 (131,585 proteins) clusters in *Z. mays* B73, and then 30,606 (83,826 proteins) clusters in AP85-441 and 25,972 (47,110 proteins) clusters in *S. bicolor*. Meanwhile, a total of 16,023 orthologous clusters (154,980 proteins) were common in four genomes. In addition, 5,884 clusters (16,654 proteins) were found to be unique to two *S. spontaneum* genomes, while 601 clusters (2,120 proteins) were identified as specific to both *Z. mays* and *S. bicolor* species. The higher number of 28,006–31,487 singletons was observed in the AP85-441 and Np-X genomes, while the lower number of 8,283 singletons was found in the Np-X genome. Furthermore, orthologous clusters among ARF gene families in four genomes were investigated (Figure 1). Overall, 17 (40 proteins), 17 (24 proteins), 21 (25 proteins), and 20 (35 proteins) orthologous ARF clusters were found in Np-X, AP85-441, *S. bicolor*, and *Z. mays* B73, respectively. Of them, a total of 16 clusters (101 proteins) of ARFs were exhibited in all four genomes, while one cluster (four proteins) was shared by two *S. spontaneum* genomes and the *S. bicolor* genome. One and seven singletons were found in *S. bicolor* and *Z. mays* B73, respectively, but none of the singletons were found in both *S. spontaneum* genomes.

### 3.2. Physiochemical and allelic information of *ARF* genes in *Saccharum spontaneum*

A total of 24 *SpapARF* and 39 *SpnpARF* sequences were found in two genomes of *S. spontaneum,* AP85-441 and Np-X, respectively. Their gene characteristics were predicted, including the length of the coding sequence (CDS), the predicted molecular weight (MW) of the protein, the isoelectric point (pI), and subcellular localization (Supplementary Table S2). For SpapARF proteins, the length of proteins ranged from 583 (*SpapARF07*) to 1904 (*SpapARF18*) amino acids (aa) and the predicted MW of both proteins ranged from 65.11 kDa to 211.59 kDa. The pI ranged from 5.28 (*SpapARF07*) to 8.30 (*SpapARF13*); the number of exons varied from 3 (*SpapARF04/15*) to 20 (*SpapARF18*); all *SpapARFs* except *SpapARF09* were predicted to be located at the nucleus. For SpnpARF proteins, the length of proteins ranged from 660 (SpnpARF14.1) to 1,647 (SpnpARF3); the predicted Mw ranged from 180.92 (*SpnpARF3*) to 73.24 (*SpnpARF6.1/7*); the pI ranged from 8.8 (*SpnpARF6.6*) to 5.83 (*SpnpARF14.1*); the exon number of *SpnpARFs* varied from 3 (*SpnpARF3/4.1/15.2/17*) to 17 (*SpnpARF22.2*). Of the 39 *SpnpARFs*, 34 genes were predicted to be located at the nucleus, while four genes were located at the chloroplast, and one gene was located at the nucleus and chloroplast.

The allelic information of *ARF* genes in two *S. spontaneum* genomes is shown in Supplementary Table S3. A total of 79 and 176 alleles were predicted in *SpapARFs* and *SpnpARFs*, respectively. The highest number of six alleles occurred in *SpapARF16*, while the lowest number of one allele/gene was found in *SpapARF12/19/20/21*. Additionally, the highest number of 15 alleles was exhibited in *SpnpARF22.2* while the lowest number of one allele/gene was found in *SpnpARF1.1/18/19*.

### 3.3. Phylogenetic analysis and gene structure and motifs identification

**Figure 2:**
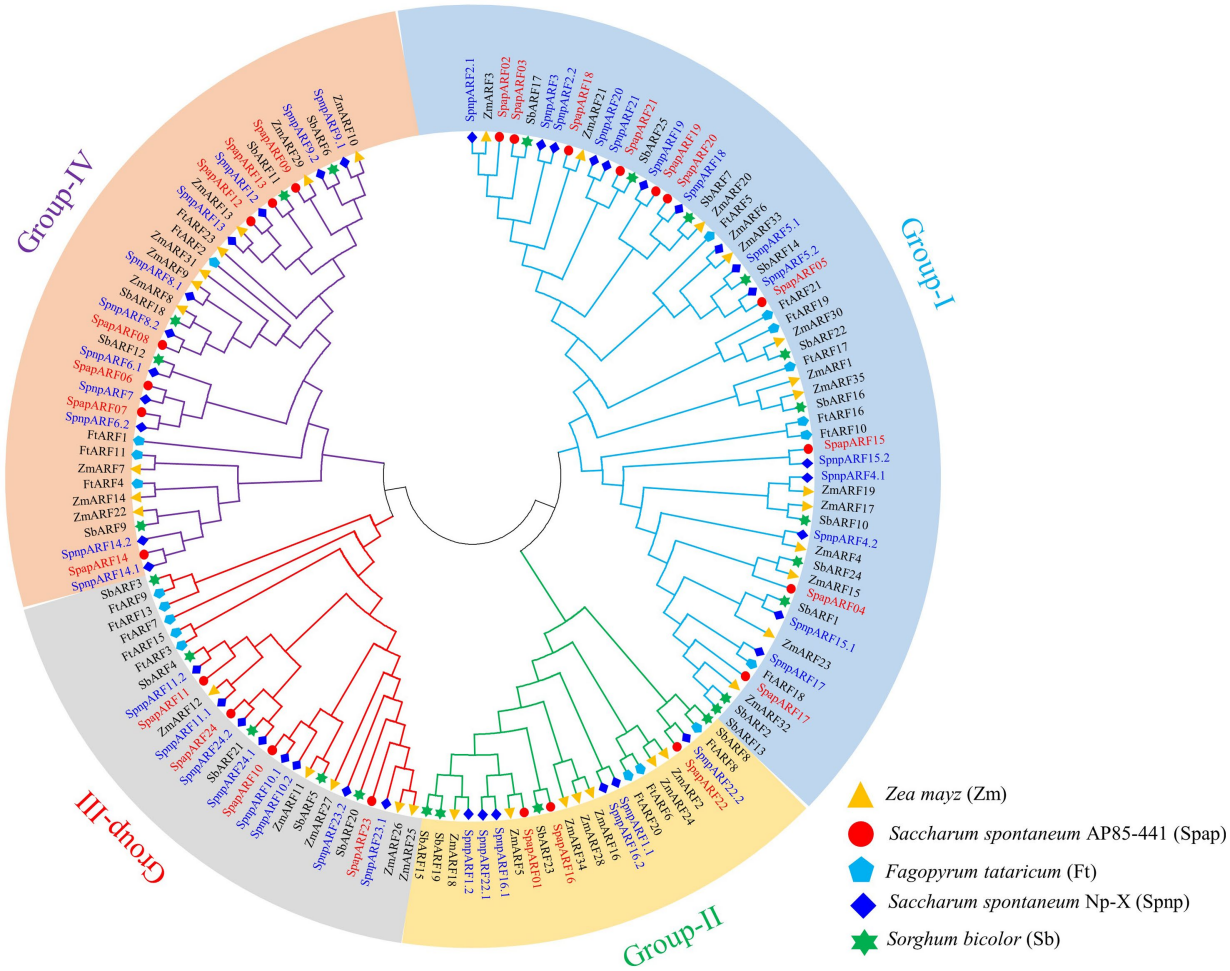
Phylogenetic tree of ARF family genes in *S. spontaneum* AP85-441 (Spap) and Np-X (Spnp), *Sorghum bicolor* (Sb), *Fagopyrum tataricum* (Ft), and *Zea mays* (Zm). This tree was constructed with the neighbor-joining method and 1,000 bootstraps implemented in the MEGA 6.0 software. Four phylogenetic Groups (I, II, III, and IV) are marked with different colored backgrounds and lines. *SpapARF* and *SpnpARF* genes are marked red and blue colors, respectively.

A phylogenetic tree was constructed using 143 ARF protein sequences, including AP85-441 (24 *SpapARFs*), Np-X (39 *SpnpARFs*), *S. bicolor* (25 *SbARFs*), *Z. mays* (35 *ZmARFs*), and *F. tataricum* (20 *FtARFs*) (Figure 2). Based on the ARF classification in maize, all ARFs were divided into four groups (I-IV). The maximum members occurred in Group-I, comprising 56 ARF members (10 *SpapARFs*, 14 *SpnpARFs*, 11 *SbARFs*, 14 *ZmARFs*, and 7 *FtARFs*), followed by Group-IV with 37 ARF members (7 *SpapARFs*, 11 *SpnpARFs*, 5 *SbARFs*, 9 *ZmARFs*, and 5 *FtARFs*), and then Group-II and Group-III, containing 23 and 27 ARF members, respectively. Furthermore, another phylogenetic tree was constructed with ARF members from two *S. spontaneum* genomes (Supplementary Figure S2). All *SpapARFs* and *SpnpARFs* were distributed in four Groups (A-D), i.e., 25 genes (10 *SpapARFs* and 15 *SpnpARFs*) in Group-A, 18 genes (7 *SpapARFs* and 11 *SpnpARFs*) in Group-B, 8 genes (3 *SpapARFs* and 5 *SpnpARFs*) in Group-C, and 12 genes (4 *SpapARFs* and 8 *SpnpARFs*) in Group-D.

**Figure 3:**
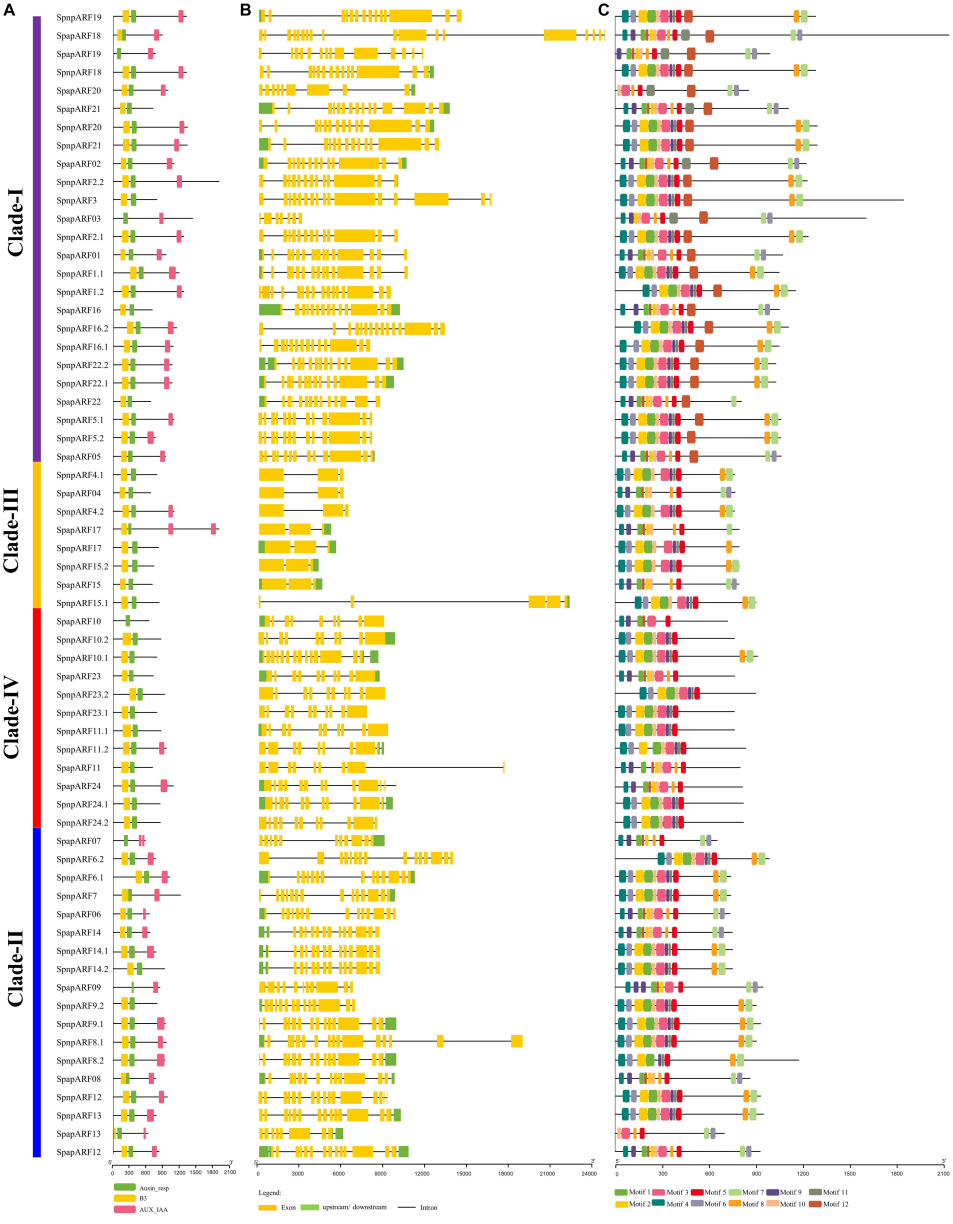
Conserved domains **(A)**, exon-intron structure **(B)**, and conserved motifs **(C)** of *ARF* genes in *S. spontaneum* AP85-441 (Spap) and Np-X (Spnp). **(A)** The Clades (I/II/III/IV) of *ARF* genes are identical to those phylogenetic groups (A, B, C, and D) in Supplementary Figure S2, respectively. The functional domain was obtained from model gene annotation and results of the NCBI CDD-batch search. B3: B3 DNA-binding domain; Auxin-resp: ARF domain; AUX_IAA: C-terminal dimerization domain. **(B)** The exons and introns in each gene are represented by lines and boxes, respectively. The sizes of exons, introns, and untranslated regions are drawn on the bottom. **(C)** Conserved motifs of *SpapARF* and *SpnpARF* proteins were identified using MEME (suite 4.11.4). Each motif is indicated with a colored box numbered 1 to 12 at the bottom. Protein length is estimated using the scale (amino acid) at the bottom in **(A)** and **(C)** panels, while gene length is estimated using the scale (bp) at the bottom in **(B)** panel.

The gene structure and motif pattern of *SpapARFs* and *SpnpARFs* were examined (Figure 3). All of them had three characteristic regions: highly conserved DBD (Pfam02362) at the N-terminal, ‘Auxin-rep’ known as the ARF domain (Pfam06507) at the middle region, and the Aux/IAA domains (CTD; Pf02309) at C-terminal (Figure 3A; Supplementary Figure S3). The DBD and “Auxin_rep” domains were present in almost all identified SpapARF and SpnpARF proteins. However, the CTD domain was absent in 20 SpapARF and SpnpARF proteins, most of which belonged to Clade-III and Clade-IV; The DBD domain was lost in five SpapARF and SpnpARF proteins, distributing in Clade-I (Ali et al., 2019), Clade-II (Ali et al., 2019), and Clade-IV (Ali et al., 2020). Notably, SpapARF07 (Clade-II) and SpapARF17 (Clade-III) had two CTD domains (Figure 2A).

Gene structure analysis demonstrated that the exon number in *ARF* genes of *S. spontaneum* ranged from three (all genes except for *SpnpARF15.1* in Clade-III) to 20 (*SpapARF18* in Clade-I; Figure 3B). In Clade-1, most *SpnpARFs* and *SpapARFs* contained 10–20 exons, except for seven exons in *SpapARF03*. *SpnpARFs* and *SpapARFs* in Clade-II contained 8–16 exons, while those *ARF* genes in Clade-IV held 8–15 exons. The majority of *ARF* genes in Clade-III had only three exons, except for *SpnpARF15.1* with five exons. The longest exon structures of *SpnpARFs* and *SpapARFs* were found in Clade-1, while the longest intron structure was observed in *SpnpARF15.1* (Clade-III). Overall, similar exon/intron numbers and their positions in *SpnpARFs* and *SpapARFs* occurred in the same clade. However, variable exon/intron numbers were present in some genes clustered in the same clade. For example, *SpnpARF15.1* in Clade-III had four introns (two longest introns) and five exons, whereas the rest of the members only had two introns and three exons.

Conserved motif analysis revealed that 12 conserved motifs (Motifs 1–12) were identified (Figure 3C), namely, Motifs 1, 2, and 10 that were annotated as DBD; Motifs 6, 8, and 12 were CTD; Motifs 3, 5, 9, and 11 were Auxin-rep, and Motifs 4 and 7 belonged to unknown domains. The length of conserved motifs varied from 15 (Motif 11) to 50 (Motifs 1–4 and 12) amino acids. Among the 12 motifs, six motifs (Motifs 1–5 and 10) were commonly found in *SpnpARFs* and *SpapARFs*. Most genes in Clade-I contained all 12 motifs, while Motifs 7 and 8 were absent in all genes except *SpnpARF10.8* in Clade-IV. However, Motif 7 was specifically present in almost all *SpnpARFs* and *SpapARFs* of Clade-I/II/III. Motif 6 was unavailable in most genes in Clade-II/III and IV.

### 3.4. Analysis of *cis*-regulatory elements in promoter regions

Twenty-two *cis*-elements responsive to biotic and abiotic stresses were predicted to be present in the 1.5 kb promoter regions of *SpapARFs* and *SpnpARFs* (Supplementary Figure S4). These elements were classified into three classes based on their functions (hormone-related, stress-related, and growth and development-related elements). Among them, 18.2% of the elements belonged to the hormone-related class, including ABRE (involved in abscisic acid responsiveness), AE-Box (light-responsive element), TGA-element (auxin-responsive element), and TGACG-motif (involved in MeJA responsiveness); 63.6% elements were related to biotic and abiotic stresses, including ARE (essential for anaerobic induction), W-box (responsive to wounding or pathogens), MYB (stress-responsive), MBS (involved in drought-inducibility), WUN-motif (wound-responsive element), as-1 (oxidative stress-responsive), DRE core (dehydration-responsive), STRE (stress-responsive), and TATA-box (stress-responsive element); 18.2% elements were associated with the development-related class, including CAAT-box (growth-related), GT1-motif (light-responsive), CAT-box (related to meristem expression), and G-box (involved in light responsiveness). Notably, *SpnpARF3* and *SpapARF15* had the highest number of 33 and 30 copies of the TATA-box element in their promotor regions, while seven genes (*SpapARF06/17/18/19* and *SpnpARF10.1/11.1/22.2*) possessed all the stress-related elements in their promoter regions. Three elements (MYC, STRE, and TATA-box) were present in all *SpapARF* and *SpnpARF* genes, while CAAT-box and MYB-binding sites specifically existed in the promoter regions of *SpapARFs* and *SpnpARFs*, respectively. All hormone-related *cis*-elements were found in the promoters of nine genes (*SpapARF02/08/17/18* and *SpnpARF1.1/3/6.1/8.2/11.1*).

### 3.5. Chromosomal location, duplication event, and synteny analysis

The distribution of *SpapARF* and *SpnpARF* genes in two *S. spontaneum* genomes (Np-X and AP85-441) is shown in Supplementary Figure S5. The majority of *SpapARF* and *SpnpARF* genes are located on the proximate or distal ends of the chromosomes. All 24 *SpapARF* genes were unevenly distributed on 16 chromosomes of AP85-441 (Supplementary Figure S5A), namely, three *SpapARFs* in chromosome Chr04B, two *SpapARFs* in six chromosomes (Chr02B, Chr03D, Chr04C, Chr05B, Chr06C, and Chr08C) each, and one *SpapARF* in nine individual chromosomes (Chr01A, Chr03B, Chr04D, Chr05A, Chr06A/D, Chr07A/C, and Chr08D). However, none of the *SpapARF* genes were distributed in the remaining 16 chromosomes of AP85-441. Meanwhile, all 39 *SpnpARF* genes were unequally distributed on 26 chromosomes of Np-X genome (Supplementary Figure S5B), i.e., four *SpnpARFs* in chromosome Chr04C, two *SpnpARFs* in 10 chromosomes (Chr03A/B, Chr04B, Chr06C, Chr07A, Chr08A/C, Chr09D, and Chr10A/D) each, and one *SpnpARF* in 15 chromosomes. However, none of the *SpnpARF* genes were found in the other 14 chromosomes of Np-X.

**Figure 4:**
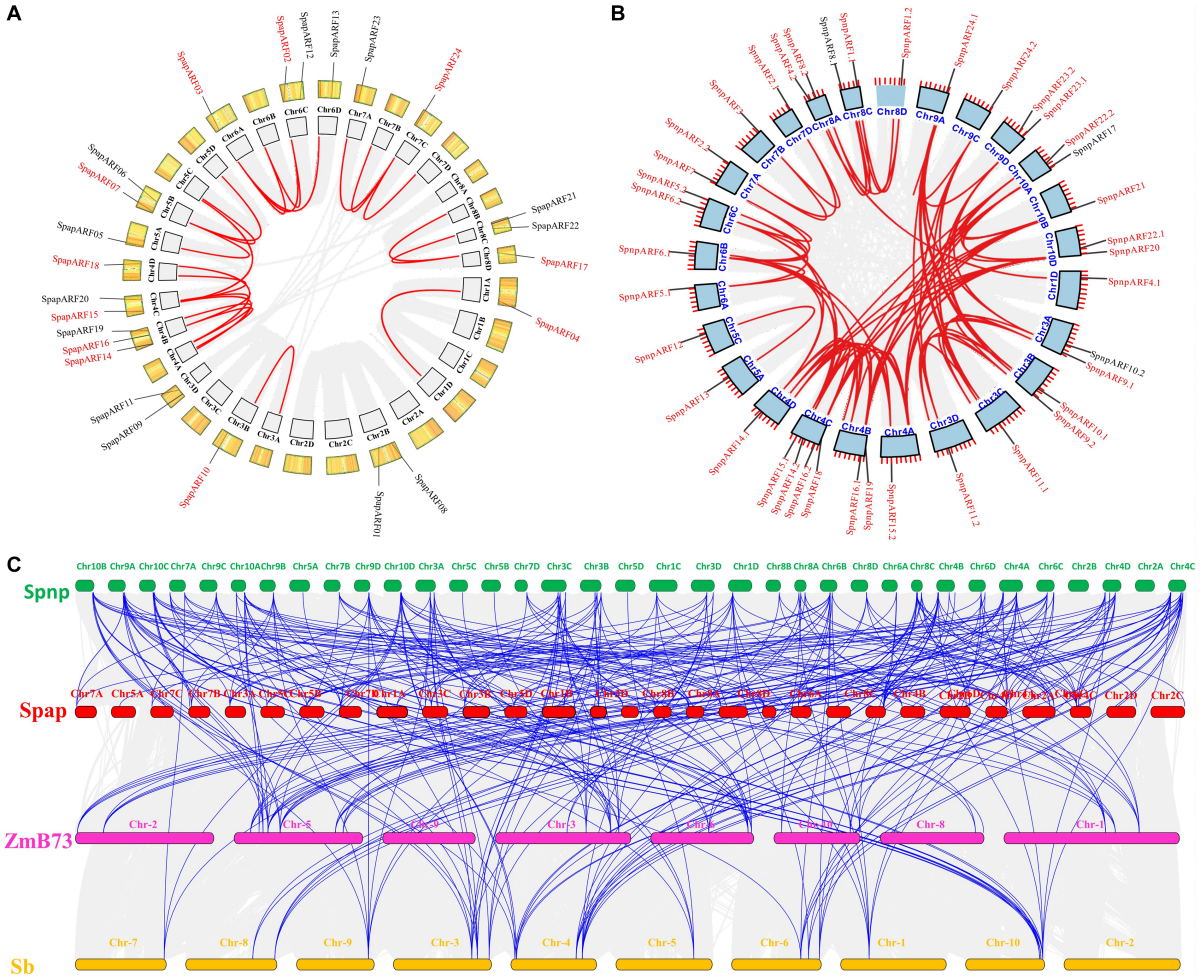
Circos illustration of duplicated *ARF* genes on chromosomes of *S. spontaneum* AP85-441 (Spap) **(A)** and Np-X (Spnp) **(B)**, and multicollinearity analysis of *ARF* genes in four genomes including two *S. spontaneum* clones, *Z. mays* B73 (ZmB73), and *S. bicolor* (Sb) **(C)**. The grey ribbon indicates a collinear relationship among blocks in the whole genome and the red ribbon shows *ARF* paralogs in **(A)** and **(B)** panels. The chromosomes are arranged in a colored arc. Genes with red color represent segmentally duplicated events. Blue lines represent syntenic *ARFs* in the **(C)** panel and gray lines in the background represent all orthologous genes among four genomes.

Collinearity and gene duplication analyses in all *SpapARFs* uncovered that 11/24 (45.8%) pairs of *SpapARF* genes were possibly involved in allelic and non-allelic segmental duplications (Figure 4A). Gene duplication was found in two pairs of non-allelic duplicated genes, including *SpapARF15*-*SpapARF16* on Chr04B-Chr04C and *SpapARF22*-*SpapARF03* on Chr06A-Chr06C. Nine allelic segmental duplications were found on different chromosomes, while seven *SpapARF* genes (*SpapARF04/07/10/14/15/16/17*) were duplicated on the unassembled scaffolds. Notably, chromosomes Chr4A/B/C/D were hotspots for gene duplication, as evidenced by four segmentally duplicated pairs. A singular duplication gene pair was identified on chromosomes Chr03A/B and Chr01A/D, while no duplicated gene pair was found on chromosomes Chr2A/B/C/D. Regarding *SpnpARF* genes in the Np-X genome, 36/39 (92.3%) gene pairs were involved in segmental duplication (Figure 4B). Eleven gene pairs including *SpnpARF-SpnpARF24.1/24.2*, *SpnpARF2.1-SpnpARF2.2-SpnpARF3*, *SpnpARF12-SpnpARF13*, *SpnpARF20-SpnpARF21*, *SpnpARF22.1-SpnpARF16.1*, *SpnpARF1.1-SpnpARF1.2*, *SpnpARF15.1-SpnpARF15.2*, *SpnpARF10.1-SpnpARF23.1-SpnpARF2.2*, and *SpnpARF4.1-SpnpARF4.2* were duplicated segmentally on various chromosomes. The remaining 25 gene pairs were segmentally duplicated on the unknown scaffold regions of different chromosomes. The most abundance of segmentally duplicated genes was present on chromosome Chr04A/B/C/D, followed by Chr03A/B/C/D and Chr10A/B/D. However, only a single duplication was observed on Chr05A/C. No tandem duplication event was present on both *S. spontaneum* genomes.

Comparative synteny analysis among *S. spontaneum* clones (Np-X and AP85-441), *Z. mays* B73, and *S. bicolor* showed that Np-X (*SpnpARFs*) and AP85-411 (*SpapARFs*) had remarkable syntenic associations with each other and *Z. mays* B73. A total of 76 *SpnpARFs* shared syntenic relationships in the AP85-441 genome, and 72 *SpnpARFs* had syntenic relationships in the *Z. mays* B73 genome. In addition, 51 *SpnpARFs* had syntenic relationships in the *S. bicolor* genome. Notably, no synthetic relationship was observed between *SpnpARF4.2* and *SpnpARF10.2* (Figure 4C; Supplementary Table S4).

### 3.6. Identification of miRNA target sites

**Figure 5:**
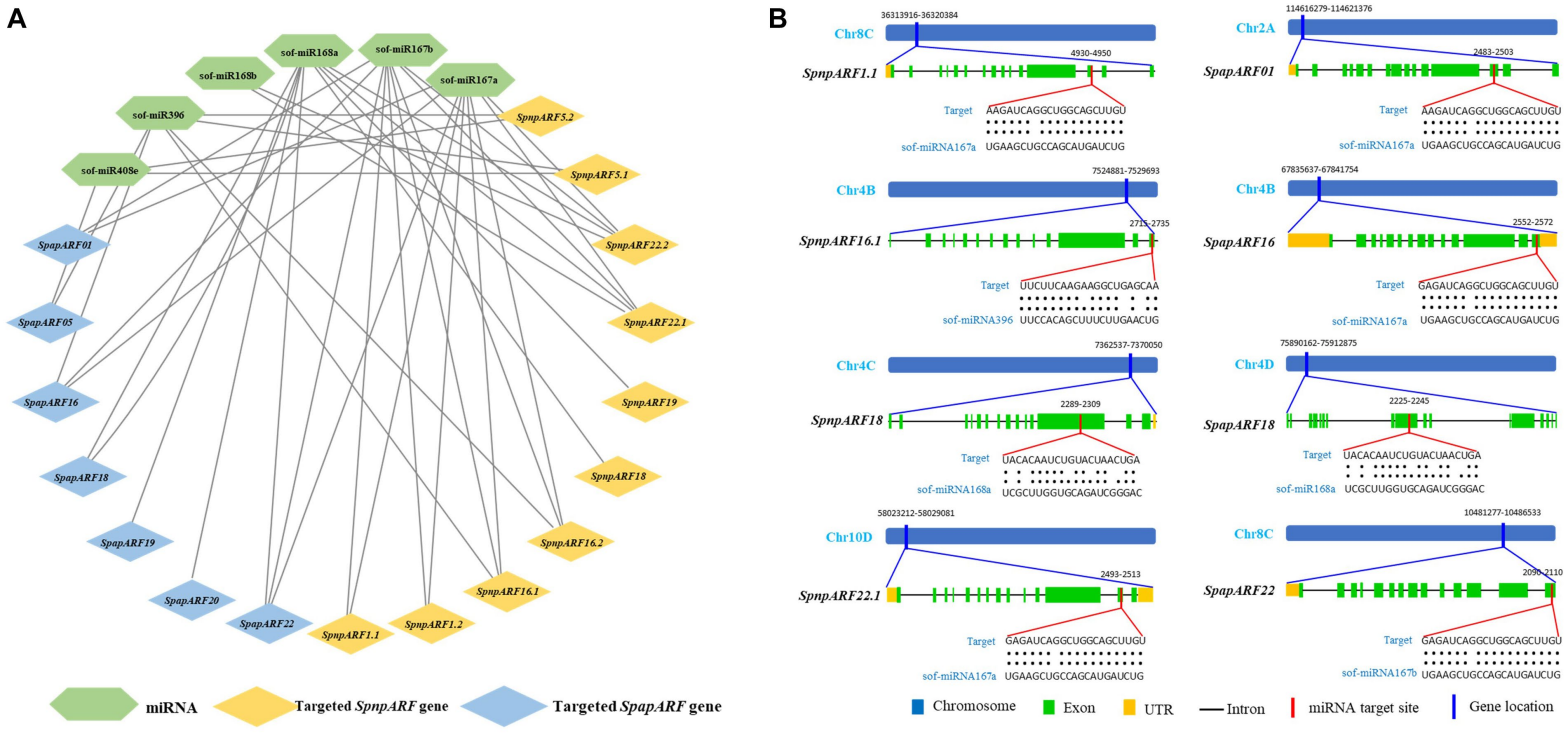
Network map of predicted miRNAs targeting *SpapARFs* and *SpnpARF*s **(A)** and schematic diagram of miRNAs targeting sites in some *ARFs* **(B)**. **(A)** Green, blue, and orange boxes stand for miRNAs and their targeted genes of *SpapARFs* and *SpnpARFs*, respectively. **(B)** The thick blue bar indicates the location of the *ARF* gene harbored at the specific chromosome. The thick red bar indicates the location of the miRNA targeted at the gene sequence. The RNA sequence of each complementary site from 5′ to 3′ and the predicted miRNA sequence from 3′ to 5′ are shown with red lines below the gene sequences. All miRNAs predicted to target *SpapARF* and *SpnpARF* genes are shown in Supplementary Table S5. Sof: *S. officinarum*.

To better understand the roles of miRNAs in managing the post-transcriptional regulation of *ARFs*, the CDS regions of 24 *SpapARFs* and 39 *SpnpARFs* were examined in the psRNATarget database against the published miRNAs of *S. officinarum*. A total of 17 genes (7 *SpapARFs* and 10 *SpnpARFs*) were targeted by six miRNAs that belong to four different families (Figure 5A; Supplementary Table S5). Two miRNAs, ‘sof-miR167a and sof-miR167b’ targeted nine genes (six *SpnpARFs* and three *SpapARFs*). The miRNA ‘sof-miR168a’ targeted nine other genes (four *SpnpARFs* and five *SpapARF*). The miRNA ‘sof-miR396’ targeted four *SpnpARFs* and two *SpapARFs*. The miRNA ‘sof-miR408e’ targeted two *SpnpARFs* and one *SpapARFs*. The miRNA ‘sof-miR168b’ targeted two *SpnpARFs*. On the other hand, *SpnpARF22.1/22.2* were targeted by four miRNAs each, while *SpnpARF16.1/16.2* and *SpapARF22* were targeted by three miRNAs each. Four genes, *SpnpARF18, SpnpARF19, SpapARF19*, and *SpapARF20*, were targeted by only one miRNA (sof-miR168a) (Figure 5A; Supplementary Table S5). The representative miRNA-targeted sites in *SpnpARF1.1/16.1/18/22.1* and *SpapARF01/16/18/22* are illustrated in Figure 5B.

### 3.7. Transcript profiling of *ARFs* in sugarcane under *Aaa* infection

**Figure 6:**
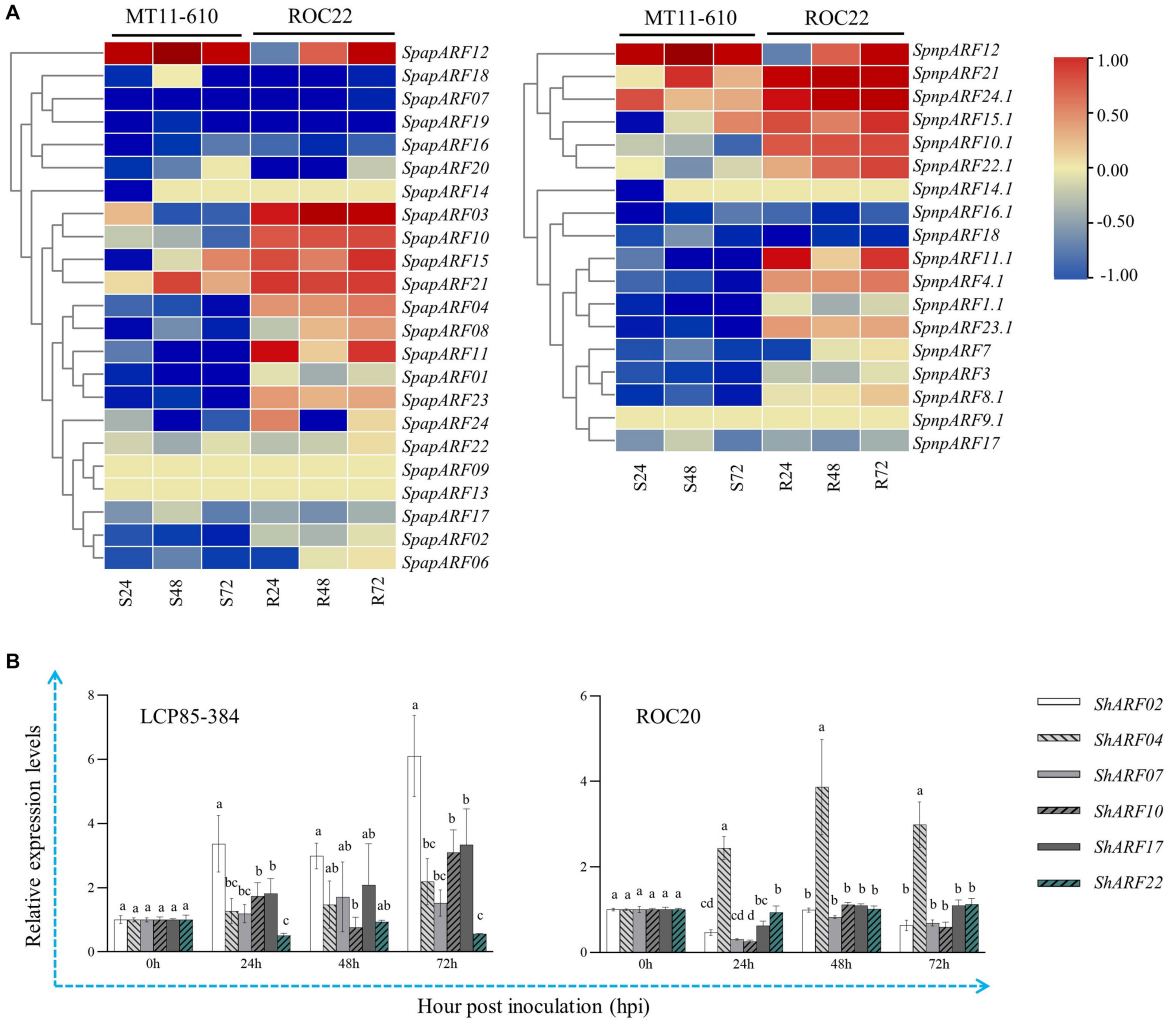
Transcript expression profiles of *SpapARF* and *SpnpARF* genes in sugarcane cultivars infected by *Acidovorax avenae* subsp*. avenae* (*Aaa*). **(A)** Transcriptome dataset of two sugarcane cultivars MT11-610 (S: susceptible to red stripe) and ROC22 (R: resistant to red stripe) triggered by *Aaa* infection. RNA-seq data were recorded for each cultivar at 0, 24, 48, and 72 h post-inoculation (hpi). Colored boxes in each column represent relative expression levels of individual genes with log_2_(Fold Change). **(B)** qRT-PCR-based expression profiling of six candidate *ARF* genes in sugarcane cultivars (ROC20 and LCP85-384) under *Aaa* infection. Leaf samples were collected at 0, 24, 48, and 72 hpi. All data are shown as the mean ± standard error (SE).

The published transcriptome dataset of MT11-610 vs. ROC22 inoculated by *Aaa* was retrieved and used to access the transcript profiles of *SpapARF* and *SpnpARF* genes (Figure 6A; Supplementary Table S6). Upon *Aaa* infection, RNA-seq data showed that eight *SpapARF* genes (*SpapARF04/10/11/23* and *SpnpARF4.1/10.1/11.1/23.1*) were upregulated in ROC22 (resistant to red stripe), while they were depressed in MT11-610 (susceptible to red stripe) across all time points. Transcript expression levels [log_2_(Fold Change)] of *SpapARF21* and *SpnpARF21/24.1* were increased in both cultivars across all time points, while their expression levels were higher in ROC22 than in MT11-610 at each time point. Meanwhile, expression levels of *SpapARF01/02/07/16/17/19* and *SpnpARF1.1/3/16.1/17/18* decreased in both cultivars across all time points. On the other hand, six representative *ShARF* genes were selected from different phylogenetic groups (*ShARF02/04/17* in Group I, and *ShARF07/10/22* in Group IV, III, and II, respectively) to analyze transcript levels in two sugarcane cultivars (LCP85-384 and ROC20) infected by *Aaa* (Figure 6B). Overall, the six genes except for *ShARF22* were upregulated to some degree in LCP85-384 at 24–72 h post inoculation (hpi). For example, the expression levels of *ShARF02/07/17* were significantly enhanced with increases of 1.2–6.1 folds in LCP85-384 but were decreased or non-significantly changed across all time points, compared with the control group (mock-inoculation at 0 hpi). *ShARF04* was positively induced in both cultivars under *Aaa* stimuli at 24–72 hpi. However, *ShARF10* was upregulated in the two cultivars at some time points, while *ShARF22* was upregulated only in ROC20 at 72 hpi, compared with the control group.

### 3.8. Transcript expression of *ARFs* in sugarcane against *Xa* infection

**Figure 7:**
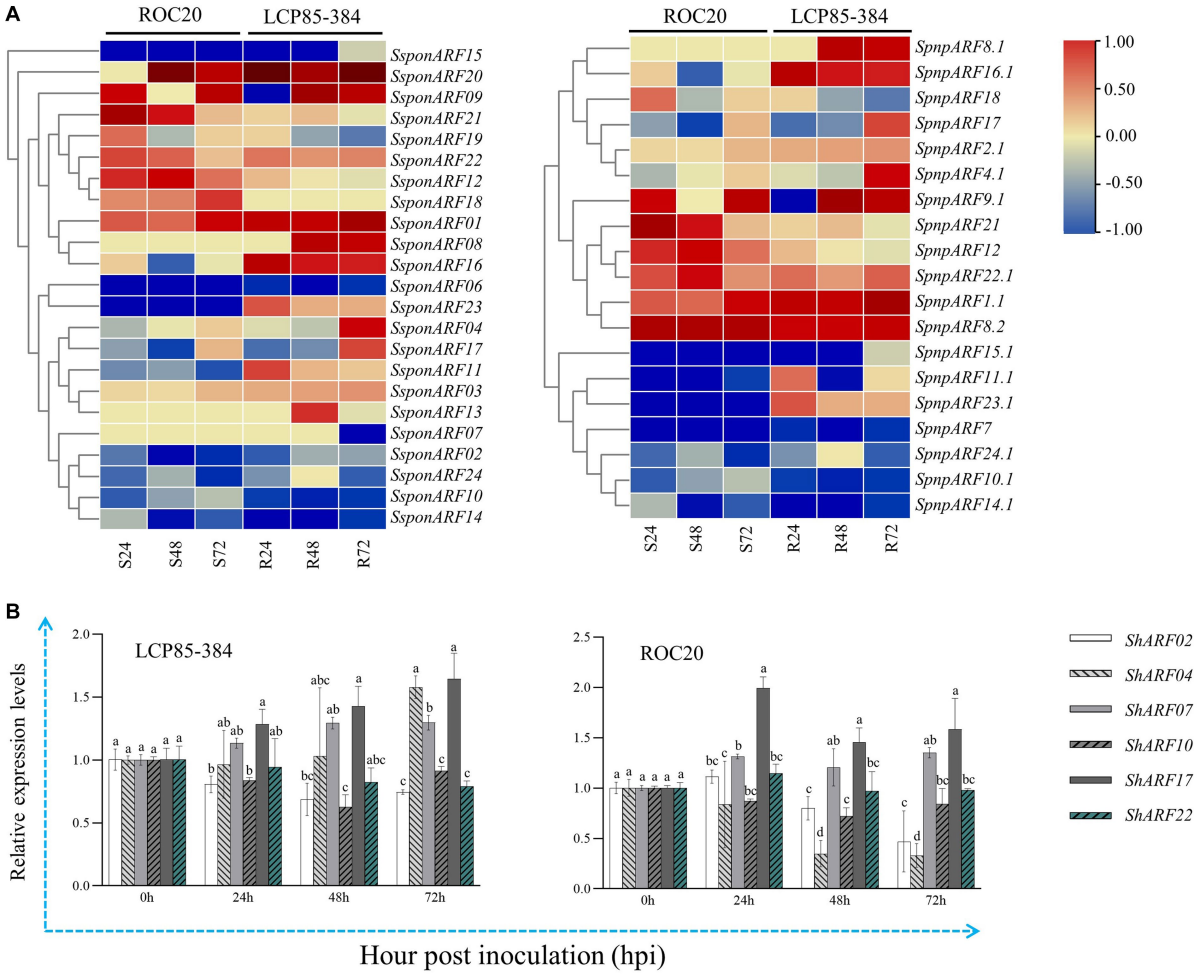
Transcript expression profiles of *SpapARF* and *SpnpARF* genes in sugarcane cultivars infected by *Xanthomonas albilineans* (*Xa*). **(A)** Transcriptome dataset of two sugarcane cultivars ROC20 (S: susceptible to leaf scald) and LCP85-384 (R: resistant to leaf scald) triggered by *Xa* infection. RNA-seq data were recorded for each cultivar at 0, 24, 48, and 72 h post-inoculation (hpi). Colored boxes in each column represent relative expression levels of individual genes with log_2_(Fold Change). **(B)** qRT-PCR-based expression profiling of six candidate *ARF* genes in sugarcane cultivars (ROC20 and LCP85-384) under *Xa* infection. Leaf samples were collected at 0, 24, 48, and 72 h post treatment (hpt). All data are shown as the mean ± standard error (SE).

Another published transcriptome dataset of LCP85-384 vs. ROC20 inoculated with *Xa* was retrieved and used to explore the transcript profiles of *SpapARF* and *SpnpARF* genes (Figure 7A; Supplementary Table S7). Eight genes (*SpapARF01/03/20/22* and *SpnpARF1.1/2.1/8.2/22.1*) were upregulated in both cultivars across all time points, but nine genes (*SpapARF02/06/10/14/15* and *SpnpARF7/10.1/14.1/15.1*) were downregulated (Figure 7A). The transcript levels of *SpapARF11/23* and *SpnpARF23.1* were increased in LCP85-384, whereas they were decreased in ROC20 in response to *Xa* infection during 24–72 hpi. Furthermore, the transcript profiles of *ShARF02/04/07/10/17/22* were identified by qRT-PCR assay in both cultivars inoculated with *Xa* (Figure 7B). Compared with mock-inoculation control, transcript levels of *ShARF07/17* were obviously increased by 13.5–64.5% in LCP585-384 and 20.7–99.2% in ROC20 at 24–72 hpi. However, *ShARF2/10/22* were downregulated in both cultivars across all time points except for a specific time point (24 hpi). *ShARF04* was significantly upregulated in LCP85-384 at 72 hpi, whereas it was dramatically depressed in ROC20 across all time points.

### 3.9. Transcript expression of *ARFs* in sugarcane under SA treatment

**Figure 8:**
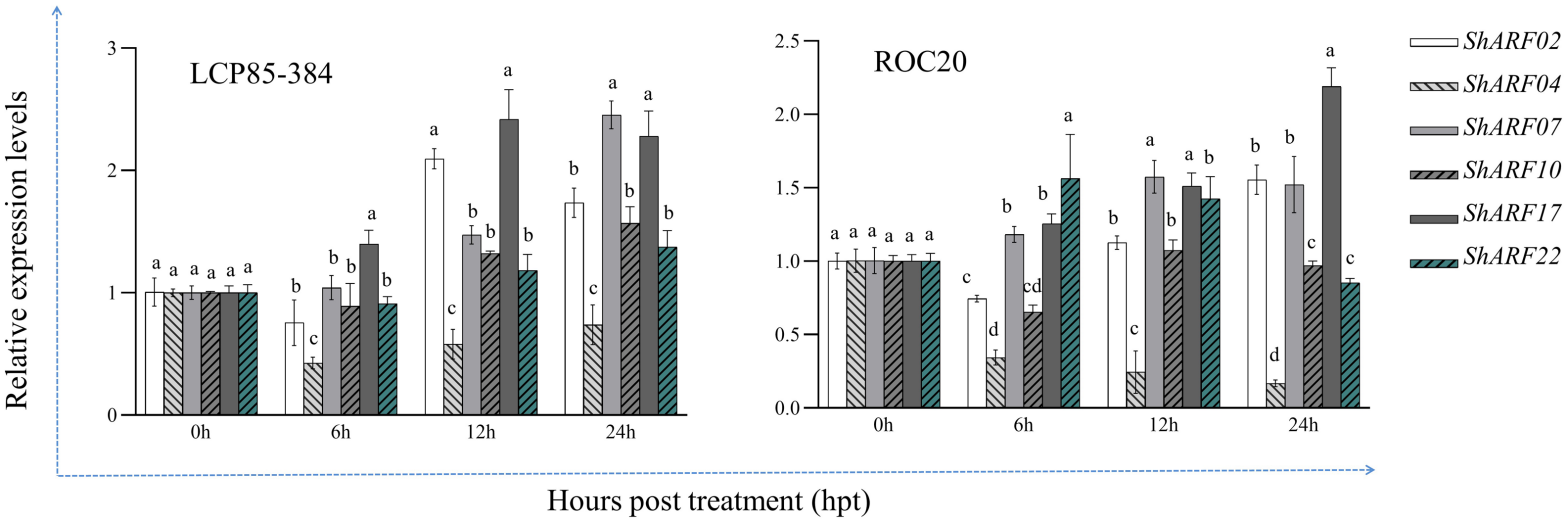
The qRT-PCR-based expression profiling of six candidate *ARF* genes in two sugarcane cultivars ROC20 (susceptible to leaf scald) and LCP85-384 (resistant to leaf scald) under exogenous salicylic acid (SA) stress. Leaf samples were collected at 0, 24, 48, and 72 h post treatment (hpt). All data are shown as the mean ± standard error (SE).

Transcript profiles of six genes (*ShARF02/04/07/10/17/22*) were explored in ROC20 and LCP85-384 under SA treatment based on qRT-PCR assays (Figure 8). Overall, expression levels of six genes except for *ShARF04* were increased in both cultivars, particularly at 12 and 24 h post-treatment (hpt). Compared with the mock-treatment (0 hpt), *ShARF02/07/17* were upregulated with increases of 1.5–2.5-fold in LCP85-384 and 1.1–2.2-fold in ROC20 at 12–24 hpt. *ShARF10* was obviously upregulated in LCP85-384 but non-significantly changed in ROC20 under SA treatment at 12–24 hpt. The expression level of *ShARF22* was significantly enhanced in LCP85-384 at 12–24 hpt and in ROC20 at 6–12 hpt. Notably, *ShARF04* was significantly decreased in both cultivars across all time points.

## 4. Discussion

An increasing number of complete sequences of plant genomes, particularly polyploid crops, is available, providing the opportunity to identify the members of the ARF gene family at the genome-wide level. Herein, we identified 24 *SpapARF* (79 alleles) and 39 *SpnpARFs* (176 alleles) family members in two genomes of *S. spontaneum* clones AP85-441 (genome size; 3.13Gb) (Zhang et al., 2018) and Np-X (genome size; 2.83 Gb) (Zhang et al., 2022). A high variation in the gene numbers of *SpapARF* and *SpnpARFs* was observed between two *S. spontaneum* genomes. This can be explained by the fact that different chromosome numbers and variations in ploidy levels are present in both *S. spontaneum* clones. AP85-441 is a haploid (1n = 4x = 32, x = 8) generated from a culture of the octoploid SES208 (2n = 8x = 64) (Zhang et al., 2018), whereas Np-X (2n = 4x = 40, x = 10) is a tetraploid (Zhang et al., 2022). The numbers of ARF family genes were obviously varied among genomes of higher plants, such as 23 in *A. thaliana* (125 Mb) (Okushima et al., 2005), and 25 ARF in *O. sativa* (450 Mb, 2n = 24) (Wang et al., 2007), 36 in *Z. mays* (2.3 Gb, 2n = 20) (Wang et al., 2012), 51 in *G. max* (978.9 Mb, 2n = 40) (Ali et al., 2022), and 67 in *T. aestivum* (17.0 Gb, 2n = 6x = 42) (Chaudhary et al., 2023). The base number of chromosomes (x), chromosome size, and ploidy level may be primary contributors to genome expansion in plants such as ferns and lycophytes (Wang et al., 2022).

Gene duplication includes whole genome duplication (polyploidization), segmental duplication, and tandem duplication, which is a very important mechanism that increases gene copy number and contributes to the generation of new genes (Goldtzvik et al., 2023). Our findings showed that fragmental duplication events of *SpapARFs* and *SpnpARFs* were detected in both genomes, especially in Np-X, but no tandem duplication event occurred, suggesting that segmental duplication events are mainly promoting the expansion of the ARF gene family. Zhai et al. (2023) also proposed that segmental duplication, rather than tandem duplications, contributed to the expansion of the ARF gene family in *Coix lacryma-jobi*. Additionally, the whole genome duplication may also contribute to this gene expansion in *S. spontaneum* due to the ploidy level of this plant species, which ranges from tetraploid (4x) to hexaploid (16x) (Zhang et al., 2022). The intron-exon patterns showed that the exon number in each *SpapARF* and *SpnpARF* gene greatly varied. Similar phenomena were reported in numerous plants, such as the dicotyledon *A. thaliana* (Okushima et al., 2005) and the monocotyledon *T. aestivum* (Chaudhary et al., 2023). These results suggested that the speciation of the ARF gene family may facilitate various biological functions across dicot and monocot plant species.

The miRNAs which are a class of small non-coding RNAs regulate gene expression through post-transcriptional modifications such as mRNA cleavage or translational inhibition, and they play critical roles in plant basic development and defense responses to environmental signals (Raza et al., 2023). In this study, six different miRNAs were predicted to target 17 *ARFs* (7 *SpapARFs* and 10 *SpnpARFs*). For instance, nine *ARFs* from *S. spontaneum* were targeted by sof-miR167a, sof-miR167b, and sof-miR168a. It seems that these *ARFs* could be regulated by sof-miR167 and sof-miR168, which may act as a crucial role in biological processes via miRNA-ARF modules involved in auxin signaling in sugarcane. A previous study showed that tobacco NtMIR167a-*NtARF6/8* was critical for tobacco regulating plant growth and inorganic phosphorus (Pi) starvation tolerance (Chen et al., 2018). Additionally, maize Zma-miR167-*ZmARF3/30* regulated polyamine oxidase 1 (*ZmPAO1*) expression and then modulated H_2_O_2_ production, conferring resistance to maize chlorotic mottle virus (Liu et al., 2022).

In this study, the temporal expression dynamics of *SpapARFs* and *SpnpARFs* were investigated in two transcriptome datasets in response to bacterial infection. Our data showed that some genes shared similar expression profiles, but some genes had different expression profiles under either *Aaa* or *Xa* infection, suggesting that sugarcane ARF family genes underwent functional divergence and redundancy in evolution. This phenomenon is frequent in plants, particularly in complex polyploidy crops such as sugarcane (Li et al., 2022). Notably, some *ARF* genes (such as *SpapARF02*) showed similar functions in sugarcane defense responses to *Aaa* and *Xa* infections, while others (such as *SpapARF01*) played the opposite role in responses to both bacterial infections. Similar appearance of functional divergence and redundancy was present in tomato *SlARFs*, as evidenced by transcript expressions of *SlARFs* in plant leaves exposed for 6 h to six biotic stressors, including flagellin, *P. putida*, *P. syringae*, *P. fluorescens*, *Agrobacterium tumefaciens*, and yellow curl virus (YCV) Bouzroud et al. (2018). For instance, *SlARF8B* (homolog of *SpapARF01*) was upregulated against *P. syringae* but downregulated under other stressors. *SlARF7B* (homolog of *SpapARF02*) was upregulated against YCV, but downregulated or unchanged under other stressors. *SlARF16A* (homolog of *SpnpARF4.1*) was downregulated in response to all biotic stress. The transcript level of *SlARF3* (homolog of *SpapARF10* and *SpnpARF10.1*) was increased under infections by *P. syringae*, *A. tumefaciens*, and YCV, while it was decreased against biotic stresses by *P. putida, P. fluorescens*, and *Flagellin* (Bouzroud et al., 2018).

It was noted that there was a discrepancy in the transcript expression profiles of six representative *ShARFs* between qRT-PCR and RNA-seq datasets, particularly in two experiments with *Aaa* infection. One reason would be that different sugarcane cultivars and *Aaa* strains were used in both datasets. For the qRT-PCR assay, leaf samples collected from two cultivars (LCP85-384 and ROC20) infected by the *Aaa* strain CNGX08 (Zhao et al., 2023) were used in this study. However, leaf samples collected from two cultivars (MT11-610 and ROC22) under the *Aaa* strain SC-026 infection (Chu et al., 2020) were used for RNA-seq. Another reason would be that these tested genes were less expressed. Everaert et al. (2017) proposed that approximately 85% of the genes showed consistent results between RNA-seq and qPCR data, and a small but specific gene set showed inconsistent results between both methods. The feature of these genes with inconsistent expression measurements was that they were typically smaller, had fewer exons, and had lower expressed levels (Everaert et al., 2017).

SA is a key plant hormone, which acts as a crucial signaling molecule for establishing defense responses in plants subjected to stressful cues (Kaya et al., 2023). Exogenous application of SA in plants can induce immune-like responses (Ding and Ding, 2020). Our results showed that *ShARF04* played a negative regulator role, whereas *ShARF07/17* acted as positive regulators under SA treatment. Similarly, *BdARF18* (homolog of *ShARF04*) was negatively induced, but *BdARF06* (homolog of *ShARF07*) and *BdARF17* (homolog of *ShARF17*) were positively regulated in *B. distachyon* in response to SA treatment (Liu N. et al., 2018). In contrast, *AcARF10* (homolog of *ShARF04*) was downregulated, but *AcARF19a* (homolog of *ShARF07*) and *AcARF26a* (homolog of *ShARF17*) were upregulated across all time points in *A. chinensis* against SA stress (Su et al., 2021). In addition, transcript levels of *ClARF5* (homolog of *ShARF04*), *ClARF* (homolog of *ShARF07*), and *ClARF7* (homolog of *ShARF17*) were upregulated by SA application during all time points in *C. lacryma-jobi* (Zhai et al., 2023). Taken together, these homologous *ARF* genes may act as positive or negative regulators against exogenous SA stress, depending on plant species. However, the regulatory mechanism of these ARFs involved in the SA-mediated plant immune response remains unclear.

## 5. Conclusion

In this study, genome-wide identification showed the presence of 24 *SpapARFs* and 39 *SpnpARFs* genes in *S. spontaneum* clones AP85-441 and Np-X, respectively. The characteristics of these ARF family genes uncovered that they had a highly divergent gene structure and a considerable number of *cis*-regulatory elements associated with biotic and abiotic stress, suggesting that these *ARF* genes possess diverse functions in sugarcane encountering environmental stress. Additionally, 17 *ARFs* from *S. spontaneum* were predicted to be targeted by numerous miRNAs, such as “sof-miR167a/b and sof-miR168a/b,” indicating that post-transcriptional regulation may participate in sugarcane in response to various stressors through the auxin signaling pathway. Furthermore, gene expression analysis revealed that redundant and divergent functions occurred among sugarcane *ARFs*, including fragmental duplication gene pairs (*ShARF17* vs. *ShARF22*) in response to bacterial infection and SA stress. Overall, this study demonstrated for the first time the genome-wide identification of the ARF gene family and its defense responses to bacterial infection and SA treatment in sugarcane, which provides an important fundament for further identification of which candidate *ARFs* are curial regulators involved in biotic and abiotic stress responses.

## Data availability statement

The data presented in the study are deposited in the NCBI repository, BioProject Accession No: PRJNA549590 (https://www.ncbi.nlm.nih.gov/bioproject/PRJNA549590) and PRJNA579959 (https://www.ncbi.nlm.nih.gov/bioproject/ PRJNA579959).

## Author contributions

J-XL: Writing – original draft. AA: Writing – review & editing. NC: Writing – review & editing and data curation. H-YF: Writing – review & editing and resources. M-TH: Resources, writing – review & editing. SM: Writing – review & editing. S-JG: Supervision, writing – review & editing, funding acquisition, and project administration. H-LZ: Writing – review & editing and supervision.

## Funding

The author(s) declare financial support was received for the research, authorship, and/or publication of this article. This work was supported by the China Agriculture Research System of MOF and MARA (grant no. CARS-170302) and the Special Projects for the Central-guided Local Science and Technology Development (grant no. 2022L3086).

## Conflict of interest

The authors declare that the research was conducted in the absence of any commercial or financial relationships that could be construed as a potential conflict of interest.

## Publisher’s note

All claims expressed in this article are solely those of the authors and do not necessarily represent those of their affiliated organizations, or those of the publisher, the editors and the reviewers. Any product that may be evaluated in this article, or claim that may be made by its manufacturer, is not guaranteed or endorsed by the publisher.

## Footnotes

^1^ http://www.life.illinois.edu

^2^ http://sugarcane.zhangjisenlab.cn

^3^ http://www.maizegenome.org

^4^ https://www.ncbi.nlm.nih.gov

^5^ http://xfam.org/

^6^ http://smart.emblheidelberg.de/

^7^ http://web.expasy.org

^8^ https://www.bioinformatics.org

^9^ https://wolfpsort.hgc.jp/

^10^ https://orthovenn2.bioinfotoolkits.net

^11^ http://gsds.gao-lab.org/

^12^ https://meme-suite.org

^13^ http://www.maizegdb.org/

^14^ https://plants.ensembl.org

^15^ http://bioinformatics.psb.ugent.be

^16^ http://www.heatmapper.ca

^17^ https://www.genscript.com
