# Supplementary figures and images for: Identification of ARF transcription factor gene family and its defense responses to bacterial infection and salicylic acid treatment in sugarcane
Source: Front Microbiol. 2023 Sep 7;14:1257355. doi: 10.3389/fmicb.2023.1257355 (PMC10513436; doi:10.3389/fmicb.2023.1257355)

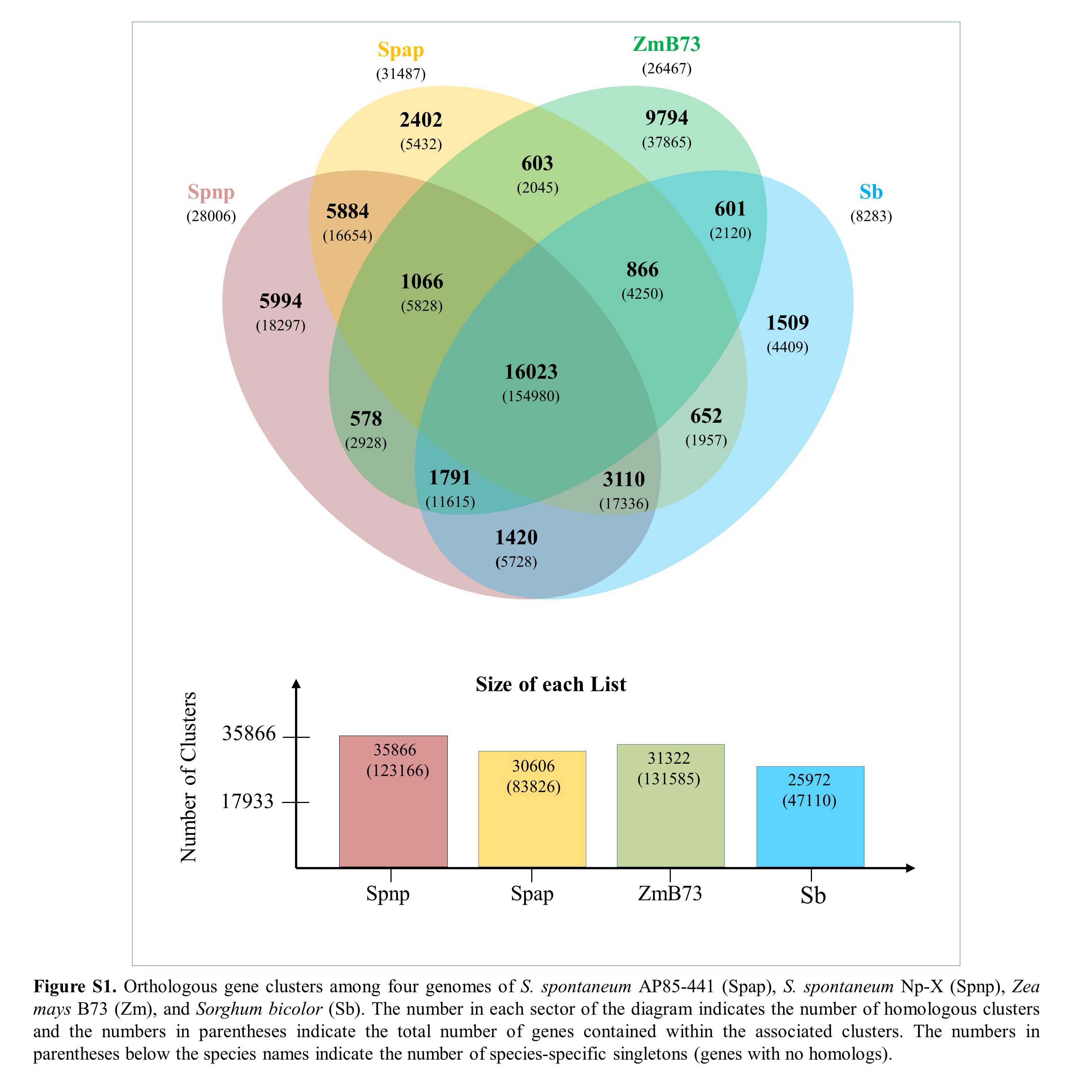

Supplement: Supplementary file 1 [file Image_1.JPEG]

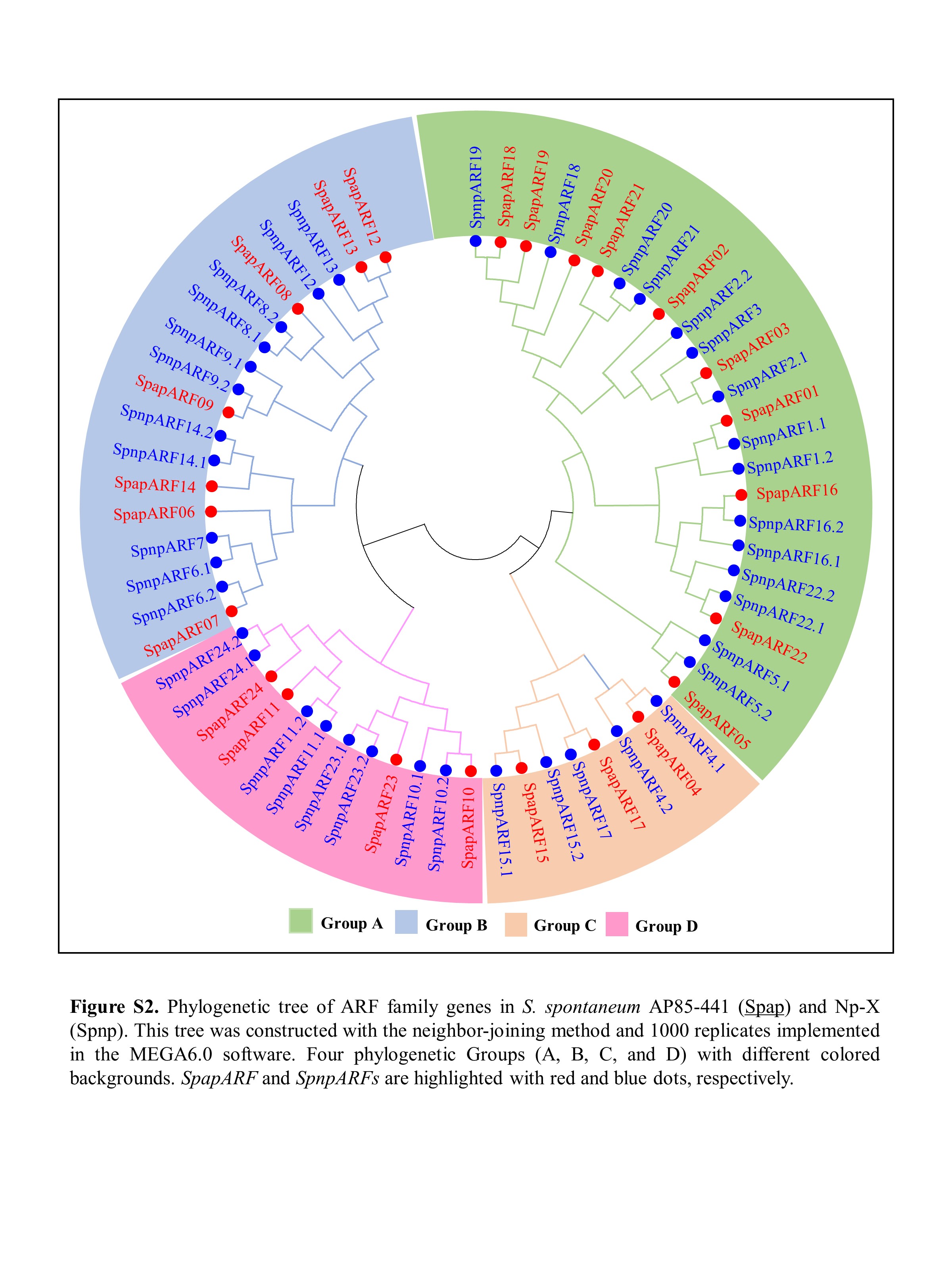

Supplement: Supplementary file 2 [file Image_2.JPEG]

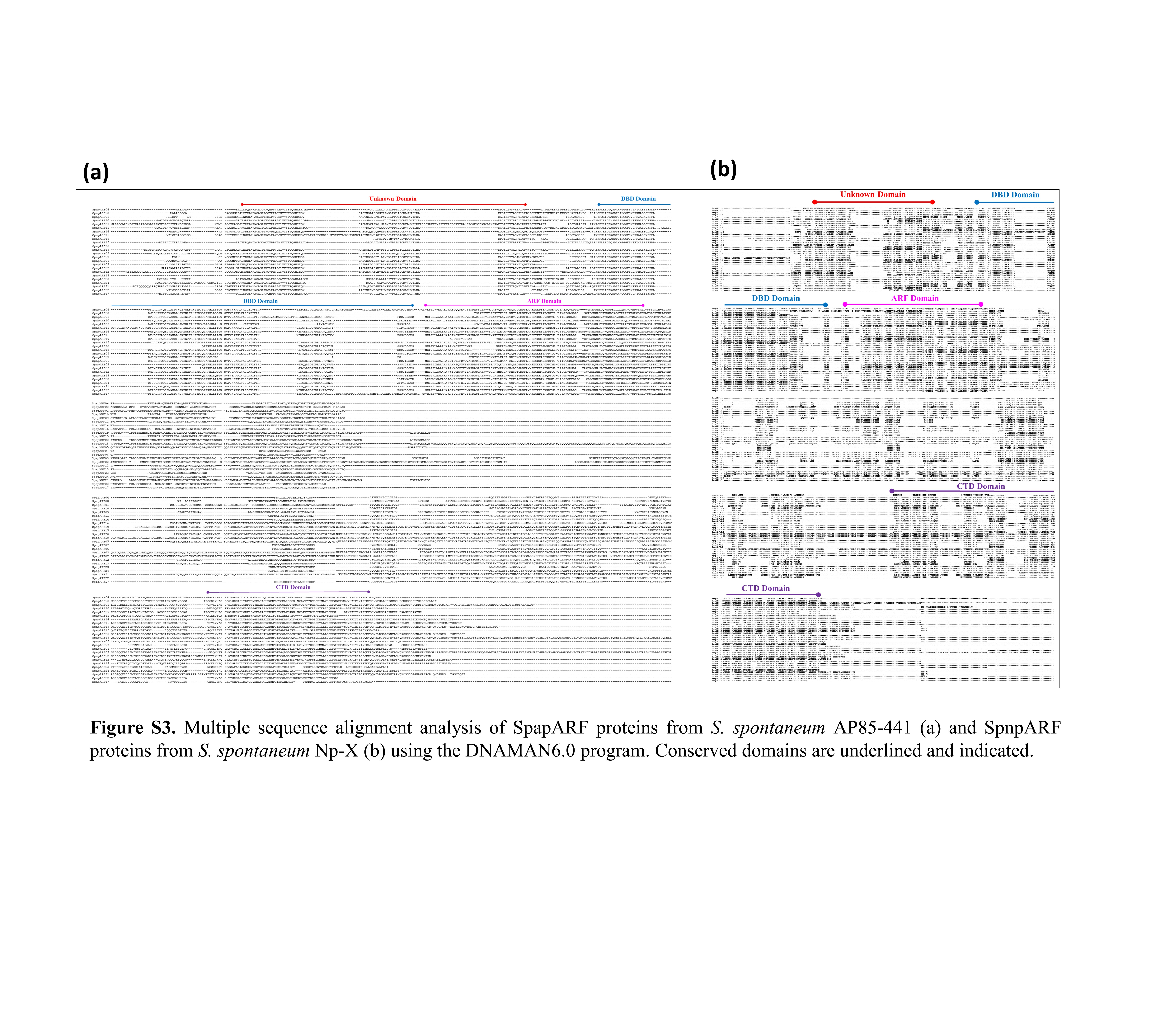

Supplement: Supplementary file 3 [file Image_3.JPEG]

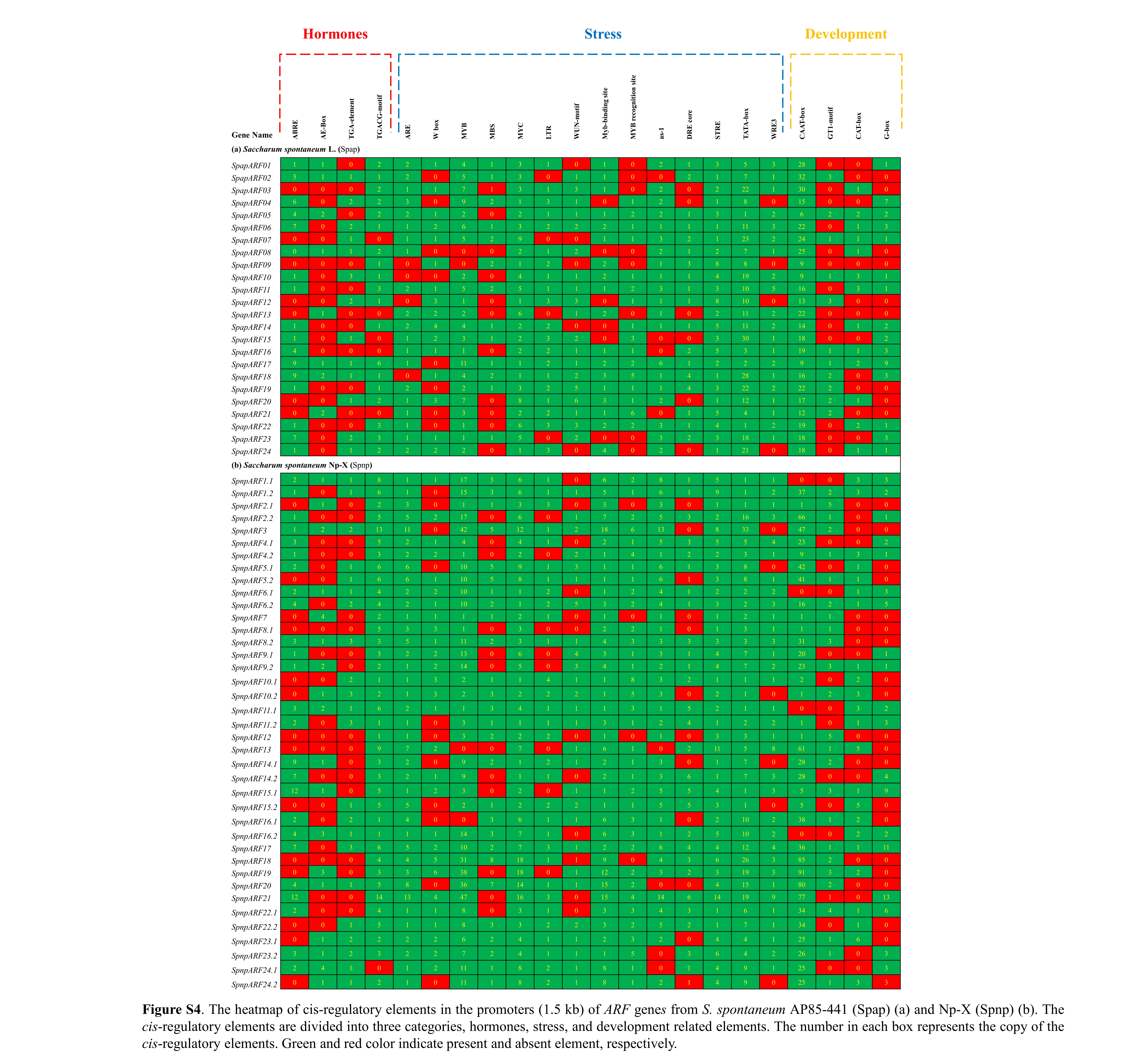

Supplement: Supplementary file 4 [file Image_4.JPEG]

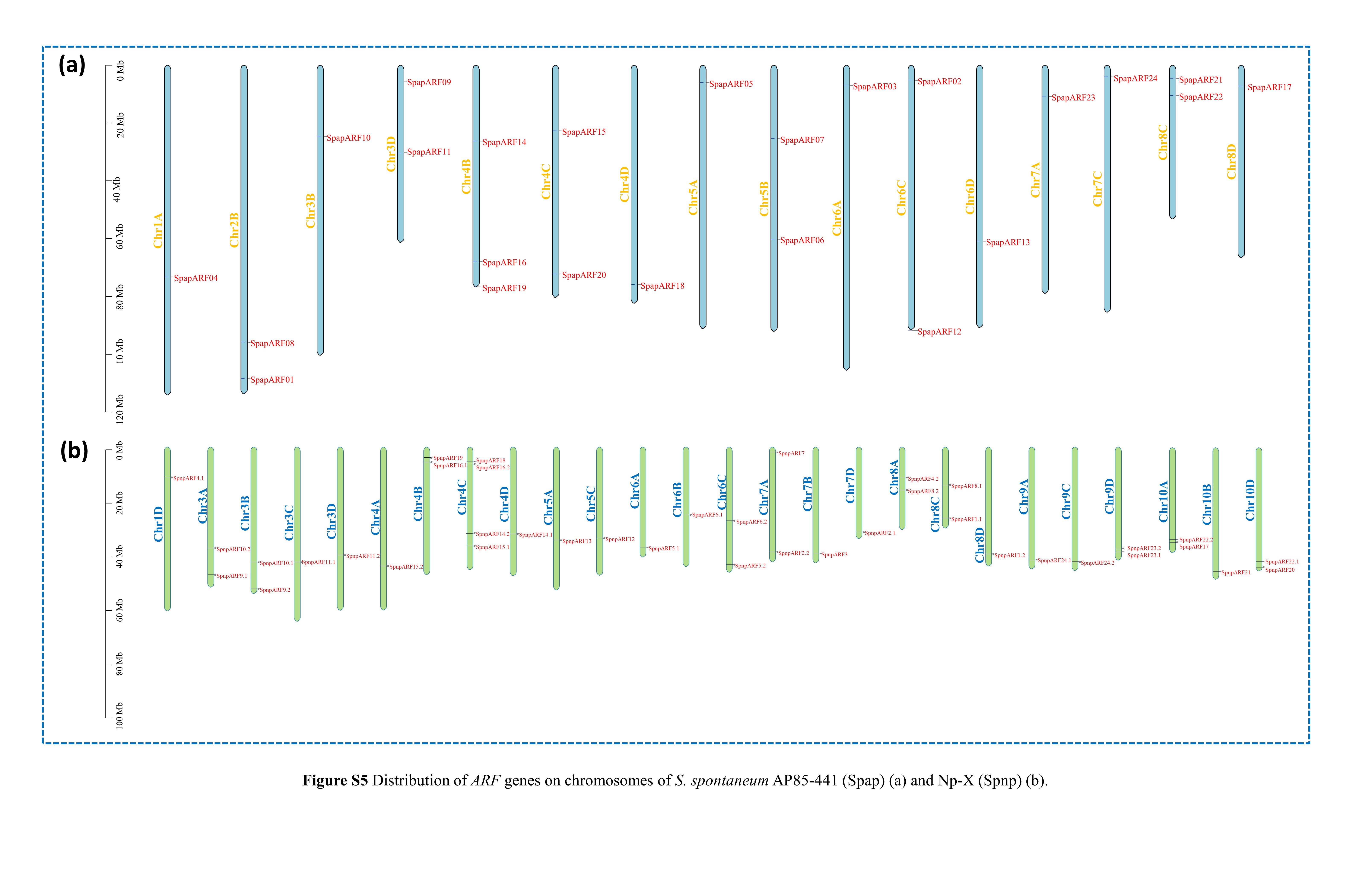

Supplement: Supplementary file 5 [file Image_5.JPEG]
